# Effectiveness of return-of-service schemes for human resources for health retention: a retrospective cohort study of four Southern African countries

**DOI:** 10.1136/bmjgh-2023-013687

**Published:** 2023-10-24

**Authors:** Sikhumbuzo A Mabunda, Andrea Durbach, Wezile W Chitha, Hawor Phiri, Mahlane Phalane, Oduetse Moaletsane, Blake Angell, Rohina Joshi

**Affiliations:** 1 School of Population Health, University of New South Wales, Sydney, New South Wales, Australia; 2 George institute for Global Health, University of New South Wales, Sydney, New South Wales, Australia; 3 Australian Human Rights Institute, University of New South Wales - Kensington Campus, Sydney, New South Wales, Australia; 4 Health Systems Enablement and Innovation Unit, University of the Witwatersrand, Johannesburg, South Africa; 5 Public Health, Walter Sisulu University, Mthatha, South Africa; 6 Mpumalanga Department of Health, Mbombela, South Africa; 7 Pharmacovigilance and Clinical Trials, Botswana Medicines Regulatory Authority, Gaborone, Botswana; 8 George Institute for Global Health, University of New South Wales, Newtown, New South Wales, Australia; 9 School of Population Health, University of New South Wales (UNSW), Sydney, New South Wales, Australia; 10 George Institute for Global Health, Delhi, India

**Keywords:** Health policy, Health systems, Public Health, Cohort study

## Abstract

**Background:**

Governments use return-of-service (RoS) schemes to train, employ and retain health professionals in the public sector. We determined the effectiveness of RoS schemes in four Southern African countries.

**Methods:**

This retrospective cohort study used databases of RoS beneficiaries from South Africa, Botswana, Eswatini and Lesotho. We ascertained the period of funding for beneficiaries between 2000 and 2010, study programme, selection criteria, study country and if they completed their studies. Records were sought to track beneficiaries’ service and fulfilment of their RoS obligations. Data were sought at the provincial level in South Africa and nationally for the other three countries. Binomial logistics regression and Kaplan-Meier survival estimates were used to determine risk factors and predictors of defaulting.

**Results:**

Most beneficiary enrolment (eg, decision on why they were funded, socioeconomic status, disability status, high school results) and service data (eg, health facilities where they worked, how long they worked at each health facility, movement between health facilities) were not available. A total of 5616 beneficiaries were drawn from the four countries’ databases. Of those with full data available, 21.7% (229/1056) were retained/served beyond their obligatory period and 20.2% (213/1056) were still serving. A total of 24.3% (95% CI: 21.7% to 26.9%; n=257/1056) of beneficiaries in the final subanalysis of two South African provinces fulfilled their contractual obligations. Only 32.2% (277/861) of beneficiaries undertook internship within their funding provinces. Governments needed to fund six beneficiaries to have one beneficiary complete their contractual obligation if they undertook internship outside their province.

**Conclusion:**

Record keeping in all countries was poor, hampering the effectiveness of RoS schemes. Of the units with full data available, the retention rate was below 25%, and internship being undertaken outside the funding province was associated with higher defaulter rates, calling for a policy overhaul.

**WHAT IS ALREADY KNOWN ON THIS TOPIC**

- Government return-of-service (RoS) schemes are used extensively by African countries to increase the pool of health professionals for a defined period.
- Despite the high cost of these schemes to low resource health systems, no low-income and middle-income country (LMIC) or African cohort studies have assessed the effectiveness of government RoS schemes.
- Outdated, high-income country studies mostly from the USA have found poor effectiveness.
- Qualitative evaluations in South Africa, Botswana, Eswatini and Lesotho have suggested that the schemes have been hampered by poor planning (not evidence based), lack of coordination, poor information systems, poor monitoring, high defaulter rates and lack of evaluations.
- Furthermore, beneficiaries’ service allocations have been reported to only be done postcompletion of their studies or internship, thus questioning the premises under which they were selected for funding. No quantitative evaluations of RoS schemes in these nations were found in the existing literature.

**WHAT THIS STUDY ADDS**

- This study is the only LMIC and African cohort study assessing the effectiveness of government RoS schemes.
- This retrospective cohort study comprehensively assessed the available data over 28.2 years (1995–2023) to ascertain effectiveness of RoS schemes.
- Up to three-quarters of beneficiaries defaulted the scheme suggesting poor return to service.
- While current policy allows beneficiaries to undertake an internship training outside their funding province, this study is the first to find that this practice is associated with higher defaulter rates which could be costly and undermine the objectives of RoS schemes.

**HOW THIS STUDY MIGHT AFFECT RESEARCH, PRACTICE OR POLICY**

- This study calls for needs and evidence-based planning, streamlined implementation and improved coordination, and improved information systems.
- We also call for a policy overhaul that has monitoring and evaluation of RoS schemes, preallocation of service area, health systems strengthening to improve internal capacity for training interns, including recommending internship to be undertaken inside the funding provinces.

## Introduction

Globally, health policy-makers and academics are grappling with sustainable strategies to address shortages and maldistribution of human resources for health (HRH).^1–4^ It is estimated that this shortfall will be at 10 million health workers required in 2030 with the worst shortages experienced in sub-Saharan Africa which has the highest burden of diseases.^5^ South Africa, Botswana, Eswatini and Lesotho have implemented return-of-service (RoS) schemes to improve health workforce capacity.^1 3 6 7^ These schemes fund undergraduate and postgraduate health professionals for a predefined period in exchange for public health sector service (mostly in underserved areas) for a fixed number of years.^1–3 6 8–10^ In South Africa, this is a year-for-year of service obligation without repayment for the financial assistance received (unless a beneficiary defaults).^1–3^ While the other three countries’ models have some similarities, the duration of service expected can be up to twice the number of years of studying support (Lesotho) with some financial repayments.^6^ These schemes aim to have certainty on the availability of health workforce in the public health sector for the contractually obligated period.^1–3 8^ In addition, they hope that health professionals will be retained for some time beyond their contractual obligation.^8^

In South Africa, RoS schemes for health professionals are administered by the nine provincial governments and their departments of health.^3^ In the other three countries, administration of the schemes is at a national level and for multiple professions, for example, Ministry of Labour for all undergraduate programmes in Eswatini, Ministry of Education in Botswana for pre-service beneficiaries, and the National Manpower Development Secretariat (NMDS) in Lesotho for all academic programmes.^1 6^ Most South African beneficiaries study in South Africa, though some medical students are trained in Cuba, and one province sends some health sciences students to Russia.^1–3^ The other three countries send RoS beneficiaries to South Africa and abroad, with some internal capacity in Botswana and limited pharmacy training in Lesotho.^1 6^

While these schemes target top academic achievers in Botswana, Eswatini and Lesotho, in South Africa, RoS schemes target top achievers from low socioeconomic backgrounds and aim to redress gender and racial imbalances where females and Africans (Blacks) were historically disadvantaged before 1994.^3^ Furthermore, these schemes aim to improve higher education access for those living with a disability.^3^ South African beneficiaries begin serving their contracts from the time they start their 1-year community service which is preceded by 2 years of internship for medical doctors and 1 year for pharmacists.^3^ This internship period can be undertaken anywhere within South Africa. All other countries expect beneficiaries to return to their countries at completion of their studies.^3 6^

Despite these schemes running for decades in these four countries, there is no evidence about their effectiveness. There is also no evidence of interconnection of RoS schemes with other HRH policies which aim to develop, attract, recruit and retain health professionals in rural and remote areas as advised by the WHO.^11^ A 2004 systematic review found eight cross-sectional studies and two cohort studies all of which were in high-income countries (HICs).^12^ A 2009 systematic review of evaluations of RoS schemes found 43 studies with 79.1% (34) in the USA and 8 spread between Japan (5), Canada (2) and New Zealand (1) and 1 descriptive study from South Africa.^8^ The South African study found that conditional RoS for HIV/AIDS was costly, with some beneficiaries defaulting their contracts.^8 9^ Other than the global shortages of cohort studies assessing effectiveness of RoS schemes, these schemes have never been evaluated within these countries.^3^ Previous qualitative studies have reported problems with the operation of the schemes including high defaulter rates, poor monitoring, poor coordination and poor planning with an inability to employ some beneficiaries after completion of studies.^3 13^

This study aimed to determine the effectiveness of RoS schemes in South Africa, Botswana, Eswatini and Lesotho by assessing the proportion of RoS beneficiaries who fulfil their contractual obligations, and to ascertain the proportion of beneficiaries who are retained in service beyond their contractual obligations. We also aimed to describe alignment of the selected beneficiaries with the RoS selection criteria.

## Methods

### Study design, participants and setting

We conducted a retrospective cohort study using RoS beneficiary data for the period 1 January 2000 to 31 December 2010 and followed them up until 2023. A detailed description of the study is described elsewhere.^1^ Briefly, in each country, beneficiary database custodians were approached for information on individuals funded between 2000 and 2010 regardless of when studies commenced or ended, as long as the funding period overlapped with this period. Subjects had to have studied for a qualification towards any one of nine possible professions, namely; medical doctor, pharmacist, dentist, physiotherapist, occupational therapist, audiologist, speech therapist, dually trained speech therapist and audiologist, and medical specialists. This cohort was followed up in two phases: first, from the commencement of their funding until completion or termination of their studies (including death); second, from the commencement of their employment until termination of service (resignation, retirement, dismissal or death) up until 31 March 2023. Databases were requested from all nine South African provincial Department of Health, Botswana Ministry of Education (preservice beneficiaries), Botswana Ministry of Health (in-service beneficiaries), Eswatini Ministry of Labour (preservice beneficiaries), Eswatini Ministry of Public Service (in-service beneficiaries) and the NMDS in Lesotho for all beneficiaries. All beneficiaries were supported with funding towards at least their tuition, accommodation, meals and books.

### Procedures

In compliance with privacy laws, identities of beneficiaries were concealed from the main research team based in Australia. Identities were only known by office-based research assistants and database custodians in the four countries (South Africa, Botswana, Eswatini and Lesotho). The in-country data custodians created two Microsoft Excel files before sending to the Australian Research team. One had the full beneficiary identifiers but with a linking random identification number designed for this research. The second file which was the one used for analysis, removed all personal identifiers and only left the randomly created numbers for linkage. For validation of sex, South African databases contained the seventh digit of the beneficiary’s national identity to check against the provided sex. Where discrepancies were identified, the identity digit was used. The database was imported into a predesigned Microsoft Access form (online supplemental appendix A) and assessed for compliance with the study’s inclusion criteria. Individuals who may have started their studies before the year 2000 but were funded in the year 2000 or after (similar to those who studied beyond 2010), were included. The database captured high school academic results, sociodemographic characteristics, family’s socioeconomic status, university details and the service record.^1^ Only two South African provinces had longitudinal service data of their Cohort. Initially, the data were 25% complete with missing service records. For beneficiaries from that province, data were exported to Excel from Microsoft Access and sent to a local Research Assistant working for the Department of Health with experience of the government payroll system known as the Personnel and Salaries Administration System (PERSAL). This researcher’s responsibility was to fill the gaps by scrutinising each individual’s movements from the first to last day of employment in that province. Even though PERSAL is used throughout all government departments in South Africa (and is the only one), it is not possible for officials in one province to monitor movement of an individual in another province. Data were then checked by the research team and the research assistant reengaged until all individuals were accounted for (a process that took three rounds of data exchange). Even though the study aimed to study all beneficiaries funded for that period (estimated to be 14 000 by key policymakers responsible for the schemes), a sample size calculation was undertaken to determine the required minimum sample size. Plans to undertake this research in Namibia were abandoned due to logistical challenges. Using the equation, $n=\frac{p\left(100-p\right)z^{2}}{d^{2}}$ for a one-sided 95% CI (z=1.96); with p (proportion of defaulters) estimated at 50% since the defaulter rate was unknown and d (desired precision) of 3% yielded a minimum sample size of 1067. We added 20% (~214) for attritions and data entry errors and this yielded a minimum sample size of 1281.

10.1136/bmjgh-2023-013687.supp1 Supplementary data

### Statistical analysis

Data were analysed using STATA version 17.0 (StataCorp). Categorical variables are summarised using graphs and frequency tables. Normality of numerical data was explored using the Shapiro-Wilk test. Since numerical variables were not normally distributed, they are summarised using the median and IQR ((IQR) or the 25th (p25) percentile and the 75th percentile (p75)). The Wilcoxon signed-rank test was used to compare the median duration of funding with the median duration of service. The median age and duration of service of defaulters and non-defaulters were compared using the Wilcoxon rank-sum test (Mann-Whitney U test). Categorical data are summarised using frequencies, percentages and graphs. Binomial logistics regression analyses were undertaken to determine the bivariate risk factors of defaulting and determined the risk ratio (RR). Similar to the calculation of the number needed-to-treat, this study calculates the number needed-to-fund for internship and community service being undertaken outside their provinces. The equation used is: $\frac{1}{\left[(riskofdefaultingifinternshipisoutsidetheprovince)-\;(riskofdefaultingifinternshipisintheprovince)\right]}$

Survival analyses were conducted using Kaplan-Meier survival estimates to determine the duration of service and fulfilment of contractual obligations. Forward selection model building criteria were used to select the best fitting Cox-proportional hazard models using the lowest Akaike information criterion (online supplemental appendix B). Model diagnostics were performed using the Cox-Snell, Martingale and Schoenfeld residuals. Hazard Ratios (HRs) were used to determine the predictors of retention. The 95% CIs are used for the precision of estimates.

10.1136/bmjgh-2023-013687.supp2 Supplementary data

### Patient and public involvement

Patients and/or the public were not involved in the design or conduct or reporting and will not be involved in the dissemination of this study as this policy is mainly relevant for health workers and policy-makers.

## Results

### Beneficiary characteristics

From January 1995 to December 2010 (16.0 years), a total of 5616 beneficiaries were drawn from the four countries’ databases as shown in tables 1 and 2). Full records were available for 19.9% (1116/5616) of the funded beneficiaries. While data were retrieved from both the Ministry of Health and the Ministry of Education in Botswana, the latter could not retrieve data of beneficiaries who transferred from the University of Botswana to South Africa for the period between 2002 and 2008. The Botswana Ministry of Health could not retrieve data for the period 2000–2002. Only Eswatini’s Ministry of Labour was able to retrieve data for the period 2000–2010, with the help of a third party hired to trace defaulters. Two South African provinces (Free State and Western Cape) could not retrieve the database for the period of assessment; three (North West, Eastern Cape (EC) and Gauteng) could only retrieve data for medical registrars (EC) and medical students who studied in Cuba; KwaZulu-Natal (KZN) could not retrieve details of beneficiaries enrolled within a South African university for the period 2000–2008. Tables 1 and 2 represent data of those beneficiaries whose details could be retrieved and not all those funded during the period of assessment. None of the countries could retrieve the decision-making tool (including school grades, presence of disability and enrolment socioeconomic status) that informed the awarding of bursary or scholarship. Eswatini is also the only country that could retrieve information on beneficiary repayment/reimbursement obligations.

**Table 1:** Basic characteristics of Botswana, Eswatini and Lesotho beneficiaries (2000–2010)

Characteristics	Overall	Botswana	Eswatini	Lesotho
	n=1257	n=311	n=110	n=836
Sex; n (%)				
Female	847 (67.4)	165 (53.1)	74 (67.3)	608 (72.7)
Male	407 (32.4)	143 (46.0)	36 (32.7)	228 (27.3)
Unknown	3 (0.2)	3 (1.0)	–	–
Degree; n (%)				
Medicine	543 (43.2)	188 (60.5)	49 (44.6)	306 (36.6)
Pharmacy	585 (46.5)	66 (21.2)	27 (24.6)	492 (58.9)
Occupational therapy	23 (1.8)	4 (1.3)	0 (0)	19 (2.3)
Dentistry	41 (3.3)	19 (6.1)	13 (11.8)	9 (1.1)
Physiotherapy	34 (2.7)	7 (2.3)	19 (17.3)	8 (1.0)
Speech and/or audiology	6 (0.5)	4 (1.3)	2 (1.8)	0 (0)
Medical specialisation	25 (2.0)	23 (7.4)	0 (0)	2 (0.2)
Country of study; n (%)				
South Africa	454 (36.1)	58* (18.7)	95 (86.4)	301 (36.0)
Botswana	6 (0.5)	6 (1.9)	0 (0)	0 (0)
Lesotho	481 (38.3)	13 (4.2)	2 (1.8)	466 (55.7)
Nigeria/Ghana†	36 (2.9)	10 (3.2)	0 (0)	26 (3.1)
Australia	17 (1.4)	17 (5.5)	0 (0)	0 (0)
Caribbean‡	185 (14.7)	167 (53.7)	0 (0)	18 (2.2)
UK	25 (2.0)	25 (8.0)§	0 (0)	0 (0)
USA/Russia¶	21 (1.7)	10 (3.2)	11 (10.0)	0 (0)
China/India**	5 (0.4)	2 (0.6)	0 (0)	3 (0.4)
Kenya/Tanzania/ Malawi/Zimbabwe/Tanzania/Morocco††	27 (2.1)	3 (0.9)	2 (1.8)	22 (2.6)
Year; n (%)				
2000–2002	138 (11.0)	34 (10.9)	26 (23.6)	78 (9.3)
2003–2005	176 (14.0)	9 (2.9)	12 (10.9)	155 (18.5)
2006–2008	411 (32.7)	140 (45.0)	36 (32.7)	235 (56.0)
2009–2010	532 (42.3)	128 (41.2)	36 (32.7)	368 (44.0)

*Excludes three individuals who split studies between South Africa and Botswana.

†(Botswana: only Ghana; Lesotho: only Nigeria).

‡Botswana: Grenada=59, Jamaica=54, Trinidad and Tobago=54; Eswatini: Cuba=18).

§Excludes one individual who split studies between Botswana and the UK.

¶(Botswana: only USA; Eswatini: only Russia).

**(Botswana: only China; Eswatini: China (2), India (1)).

††(Only Kenya in Botswana; Eswatini: Tanzania and Morocco (1 each); Lesotho: Kenya (2), Zimbabwe (14), Malawi (6)).

**Table 2:** Basic characteristics of South African beneficiaries (1995–2010) by province

Characteristics	Overall (n=4359)	EC (n=164)	LP (n=2565)	GP (n=30)	KZN (n=347)	MP (n=1017)	NC (n=99)	NW (n=137)
Sex; n (%)								
Female	2004 (46.0)	48 (29.3)	1 210 (47.2)	13 (43.3)	137 (39.5)	488 (48.0)	49 (49.5)	59 (43.1)
Male	2285 (52.4)	104 (63.4)	1 355 (52.8)	16 (53.3)	153 (44.1)	529 (52.0)	50 (50.5)	78 (56.9)
Not stated	70 (1.6)	12 (7.3)	–	1 (3.3)	57 (16.4)	–	–	–
Country of study; n (%)								
South Africa	3676 (84.3)	57 (34.8)	2403 (93.7)	0 (0)	205 (59.1)	962 (94.4)	49 (49.5)	0 (0)
Cuba	685 (15.7)	107 (65.2)	162 (6.3)	30 (100.0)	142 (40.9)	57 (5.6)	50 (50.5)	137 (100.0)
Race; n (%)*								
African	4047 (96.2)	126 (77.8)	2 560 (99.8)	29 (96.7)	185 (90.2)	955 (94.2)	57 (61.3)	135 (98.6)
Mixed race	47 (1.1)	5 (3.1)	1 (0.0)	0 (0)	2 (1.0)	6 (0.6)	31 (33.3)	2 (1.5)
White	76 (1.8)	25 (15.4)	2 (0.1)	1 (3.3)	1 (0.5)	42 (4.1)	5 (5.4)	0 (0)
Indian	36 (0.9)	6 (3.7)	2 (0.1)	0 (0)	17 (8.3)	11 (1.1)	0 (0)	0 (0)
Age, years; median (p25–75)†	20 (19–23)	20 (19–22)	21 (19–23)	–	20 (19–21)	20 (19–22)	20 (19–21)	20 (19–22)
Age categories, years; n (%)†								
<25	3688 (86.9)	102 (96.2)	2183 (85.1)	–	334 (96.3)	877 (86.1)	64 (88.9)	128 (93.4)
≥25	558 (13.1)	4 (3.8)	382 (14.9)	–	13 (3.8)	142 (13.9)	8 (11.1)	9 (6.6)
Degree; n (%)								
Medicine	2735 (62.7)	107 (65.2)	1494 (58.3)	30 (100.0)	275 (79.3)	612 (60.2)	80 (80.8)	137 (100.0)
Pharmacy	695 (15.9)	0 (0)	465 (18.1)	0 (0)	35 (10.1)	184 (18.1)	11 (11.1)	0 (0)
Occupational Therapy	250 (5.7)	0 (0)	166 (6.5)	0 (0)	12 (3.5)	69 (6.8)	3 (3.0)	0 (0)
Dentistry	246 (5.6)	0 (0)	165 (6.4)	0 (0)	8 (2.3)	70 (6.9)	3 (3.0)	0 (0)
Physiotherapy	264 (6.1)	0 (0)	193 (7.5)	0 (0)	12 (3.5)	57 (5.6)	2 (2.0)	0 (0)
Speech and hearing therapy	111 (2.6)	0 (0)	82 (3.2)	0 (0)	5 (1.4)	24 (2.4)	0 (0)	0 (0)
Medical specialisation	58 (1.3)	57 (34.8)	0	0 (0)	0	1 (0.1)	0 (0)	0 (0)
Year**; n (%)								
1995–2002	1028 (23.6)	35 (21.3)	579 (22.6)	30 (100.0)	85 (24.5)	221 (21.7)	7 (7.1)	71 (51.8)
2003–2005	845 (19.4)	50 (30.5)	516 (20.1)	0 (0)	0 (0)	239 (23.5)	25 (25.3)	15 (11.0)
2006–2008	1569 (36.0)	52 (31.7)	1118 (43.6)	0 (0)	28 (8.1)	339 (33.3)	22 (22.2)	10 (7.3)
2009–2010	917 (21.0)	27 (16.5)	352 (13.7)	0 (0)	234 (67.4)	218 (21.4)	45 (45.5)	41 (29.9)

**Includes beneficiaries whose funding went beyond 2010

*One hundred and fifty-three subjects missing.

†One hundred and fifteen subjects missing.

EC, Eastern Cape province; LP, Limpopo province; GP, Gauteng province; KZN, KwaZulu-Natal province; MP, Mpumalanga province; NC, Northern Cape province; NW, North West province.; .

Females accounted for 53.1%, 67.3% and 72.7% of beneficiaries and most beneficiaries from Botswana, Eswatini and Lesotho studied in the Caribbean (53.7%), South Africa (86.4%) and Lesotho (55.7%), respectively. Most beneficiaries in Botswana (60.5%) and Eswatini (44.6%) were enrolled for medicine (table 1). South African beneficiaries accounted for 77.6% (4359/5616) of the total sample (median age=20 years; IQR=4); 84.3% studied within South Africa and studied medicine (62.7%); mostly African (96.2%) and male (52.4%) (table 2).

### Early and late attritions

In South Africa and Eswatini (the only countries with data), commonly encountered early attritions (1.6% or 70/4245) were of two types; namely, undergraduate beneficiaries who died during their studies (0.5% or 8/1682) and those who were academically excluded (failed their studies). Academic exclusions accounted for 4.9% (50/1017), 3.0% (3/99), 0.3% (8/2565 and 1/347) of total beneficiaries for Mpumalanga (MP), Northern Cape, Limpopo and KZN provinces, respectively. Late attritions include death in service for KZN (1.4% or 5/347) and MP (0.3% or 3/1017) beneficiaries; ill health incapacity in MP (0.4%, 4/1017); dismissal for misconduct in MP (0.1% or 1/1017) and falling out with supervisor in the Northern Cape (1.0% or 1/99); and 1.8% (2/110) who were unemployed in Eswatini.

### RoS beneficiaries with complete service records

**Figure 1:**
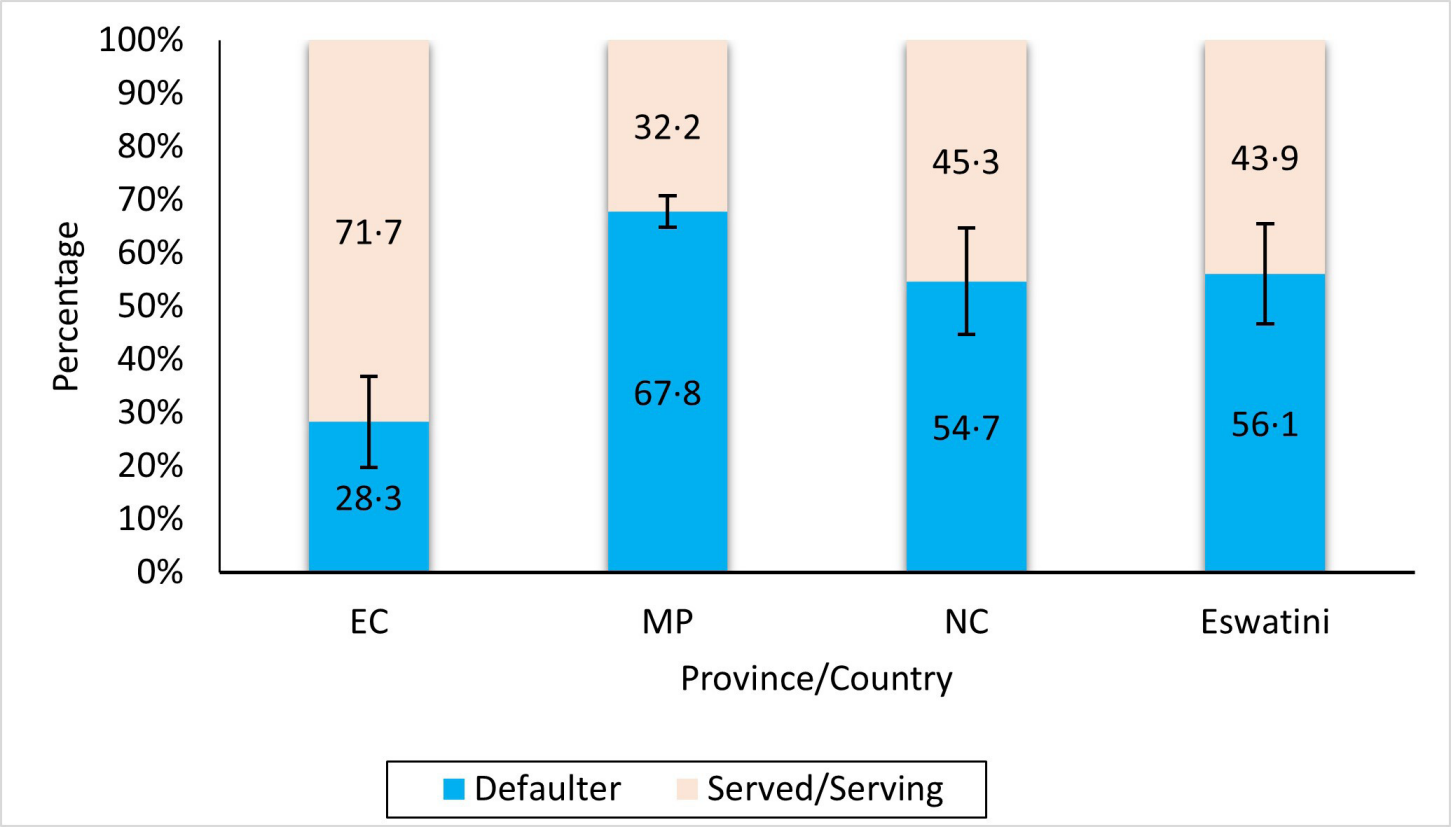
Beneficiary service compliance. EC, Eastern Cape province; MP, Mpumalanga province; NC, Northern Cape province.

Only 19.9% (1116/5616) of the data from MP and Northern Cape had complete service records and data on funding duration. While Eswatini and the Eastern Cape province had partial data on service compliance, all the other provinces and countries did not have any data on service compliance, placement and duration. After removal of the early attritions (58/5616, 1.0%), one medical specialist from MP, and one individual who was still funded and has been enrolled for studies since January 2009 (14.2 years); 18.8% (1056/5616) of beneficiaries were followed up for the second phase from 1 January 2001 to 31 March 2023 (22.2 years). Eswatini and the EC had data on defaulters but not on service (figure 1). The defaulter rate ranged from 28.3% (95% CI 19.7% to 36.9%; n=30/106) in EC to 67.8% (95% CI 64.9% to 70.8%; n=652/961) in MP and this difference between the two provinces was statistically significant (p<0.0001).

### Funding duration and service duration

The funding duration ranged from 1 to 12 years (longer for medical students studying in Cuba) and the average funding duration was 5.2 years (SD=2.0 years). The median duration of funding support (5.0 years; p25–p75=4–6 years) was statistically higher than the median duration of service (1.7 years; p25–p75=0–5.2 years; p<0.0001). In the Northern Cape and MP provinces, defaulters were likely to never serve at all, or serve a single year of their contracts.

### RoS defaulters and contractual compliance

**Figure 2:**
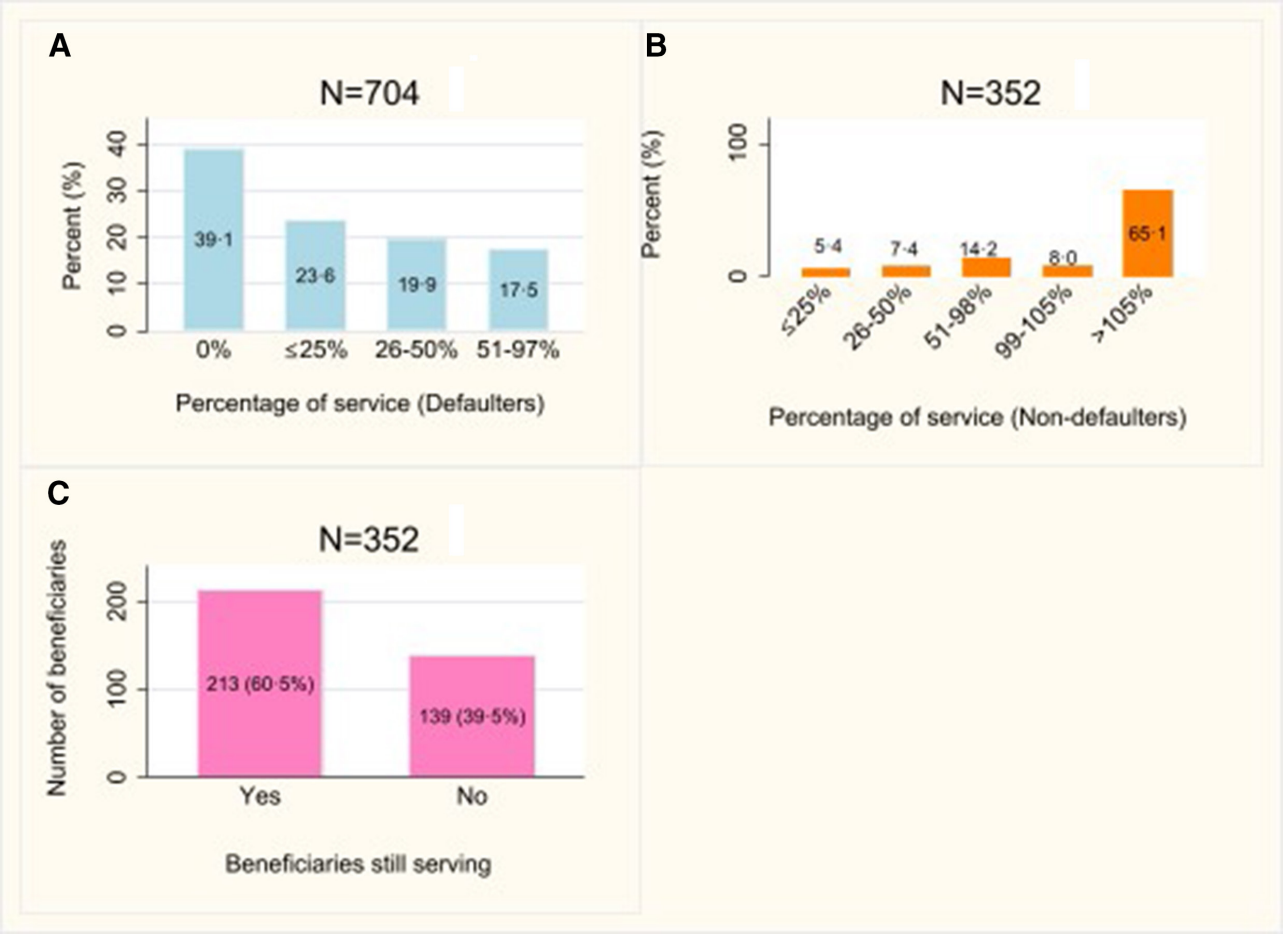
Proportion of RoS defaulters (A), non-defaulters (B) and number of beneficiaries who were serving on 31 March 2023 (C). RoS, return-of-service.

Defaulters accounted for two-thirds of the final subanalysis (704/1056; 66.7%; 95% CI 63.8% to 69.5%) or 63.1% (95% CI 60.3% to 65.9%; n=704/1116) of the initial subanalysis for the two concerned South African provinces. Wherein, the initial and final subanalyses refer to data of two South African provinces with full-service records before and after removal of early attritions and a medical specialist. Beneficiaries who fulfilled their contractual obligation were 24.3% (95% CI 21.7% to 26.9%; n=257/1056) of the final subanalysis or 23.0% (95% CI 20.6% to 25.5%; n=257/1116) of the initial subanalysis for the two provinces. Figure 2A shows that 39.1% (275/704) of beneficiaries defaulted without ever serving and, 23.6% (166/704) served no more than 25% of their contractual obligation. While 5.4% (19/352) of beneficiaries who served or were serving at the time of this study were early in their service fulfilment (≤25% served), 65.1% (229/352) served beyond their obligation (figure 2B and 60.5% (213/352) were still serving at study closure figure 2C. Those who were still serving but early in their service could still default their contracts.

### Bivariate predictors of defaulting

Defaulters were statistically more likely to be medical doctors when compared with dentists (RR=1.1; p=0.021; 95% CI 1.1 to 2.1); to have studied within South Africa (RR 2.2; 95% CI 1.8 to 2.8; p<0.0001); and to be white (RR 2.3; 95% CI 1.2 to 4.4; p=0.011) or African (RR 1.5; 95% CI 1.1 to 2.1; p=0.020) as opposed to being of mixed-race origin (table 3). Furthermore, those who undertook internship and community service outside their province were 60% (RR 1.6; 95% CI 1.3 to 2.0; p<0.0001) and 14-fold (RR 13.9; 95% CI 7.3 to 26.5; p<0.0001), respectively, more likely to default their contracts and these were statistically significant. These translate to a number needed-to-fund (NNF) of 6.1 for internship and 2.4 for community service, suggesting that almost six beneficiaries needed to be funded to have one beneficiary returning to complete their contractual obligation if they undertook internship outside their province. In comparison, approximately two individuals had to be trained for the fulfilment of RoS obligations if internship was undertaken within the province (57.0% or 158/277).

**Table 3:** Bivariate risk factors of defaulting RoS schemes in South Africa**

Characteristics	Overall	Left prematurely	Served/still serving	RR (95% CI)	P value
Service status; n (%)	1056 (100.0)	704 (66.7)	352 (33.3)	–	<0.0001*
Sex; n (%)					
Male	539 (100.0)	351 (65.1)	188 (34.9)	ref	ref
Female	517 (100.0)	353 (68.3)	164 (31.7)	1.1 (0.9 to 1.3)	0.277
Age†, years; med (p25–p75)	25.3 (23.9–28.0)	25.2 (23.9–27.8)	25.9 (23.8–28.3)	–	0.245‡
Race; n (%)					
Mixed-race	36 (100.0)	18 (50.0)	18 (50.0)	ref	ref
African	955 (100.0)	636 (66.6)	319 (33.4)	1.5 (1.1 to 2.1)	0.020
White	46 (100.0)	36 (78.3)	10 (21.7)	2.3 (1.2 to 4.4)	0.011
Indian	10 (100.0)	7 (70.0)	3 (30.0)	1.7 (0.6 to 4.5)	0.317
Degree; n (%)					
Dentistry	68 (100.0)	38 (55.9)	30 (44.1)	ref	ref
Medicine	658 (100.0)	452 (68.7)	206 (31.3)	1.4 (1.1 to 1.9)	0.021
Pharmacy	189 (100.0)	121 (64.0)	68 (36.0)	1.2 (0.9 to 1.7)	0.223
Physiotherapy	53 (100.0)	35 (66.0)	18 (34.0)	1.3 (0.8 to 2.1)	0.266
Occupational therapy	65 (100.0)	41 (63.1)	24 (36.9)	1.2 (0.8 to 1.8)	0.401
Speech therapy and/or audiology	23 (100.0)	17 (73.9)	6 (26.1)	1.7 (0.8 to 3.5)	0.163
Country of study; n (%)					
Cuba	100 (100.0)	41 (41.0)	59 (59.0)	ref	ref
South Africa	558 (100.0)	411 (73.7)	147 (26.3)	2.2 (1.8 to 2.8)	<0.0001
Internship in funding province§; n (%)					
Yes	277 (100.0)	158 (57.0)	119 (43.0)	ref	ref
No	584 (100.0)	429 (73.5)	155 (26.5)	1.6 (1.3 to 2.0)	<0.0001
Community service in funding province; n (%)					
Yes	774 (100.0)	431 (55.7)	343 (44.3)	Ref	ref
No	282 (100.0)	273 (96.8)	9 (3.2)	13.9 (7.3 to 26.5)	<0.0001
RoS fulfilment, years; med (p25–p75)					
Medicine	1.9 (1.0–4.7)	1.0 (0–2.1)	6.0 (4.0–8.9)	–	<0.0001‡
Pharmacy	1.3 (0–6)	1.0 (0–1.3)	9.2 (5.4–11.6)	–	<0.0001‡
Dentistry	3.0 (0–10.4)	1.0 (0–1.0)	10.8 (7.6–12.2)	–	<0.0001‡
Physiotherapy	1.0 (0–6.7)	1.0 (0–1.0)	8.5 (6.7–13.2)	–	<0.0001‡
Occupational therapy	1.6 (0–6.1)	0.0 (0–1.2)	8.0 (6.0–10.7)	–	<0.0001‡
Speech therapy and/or audiology	2.2 (0–3.9)	2.0 (0–2.3)	4.7 (4.0–10.2)	–	0.004‡
All programmes	1.7 (0–5.2)	1.0 (0–2.0)	7.0 (4.7–10.2)	–	0.0001¶
RoS fulfilment, years; n (%)					
5–7	134 (100.0)	26 (19.4)	108 (80.6)	ref	ref
None	275 (100.0)	275 (100.0)	0 (0.0)	–	–
One	230 (100.0)	219 (95.2)	11 (4.8)	16.9 (9.4 to 30.2)	<0.0001
2–4	257 (100.0)	182 (70.8)	75 (29.2)	2.8 (2.2 to 3.4)	<0.0001
8–9	58 (100.0)	2 (3.4)	56 (96.6)	0.8 (0.8 to 0.9)	<0.0001
10–21	102 (100.0)	0 (0.0)	102 (100.0)	–	–

*Two-sample test of proportions was used.

†Age at study completion.

‡Wilcoxon rank-sum test was used.

§Only pharmacists and medical doctors.

¶Kruskal-Wallis test was used.

**Data exclude early attritions; one medical specialist in MP and the one individual who is still studying.

med, median; p25, 25th percentile; p75, 75th percentile; ref, reference; RoS, return-of-service; RR, risk ratio.

### Survival analysis and Cox-proportional hazard models

**Figure 3:**
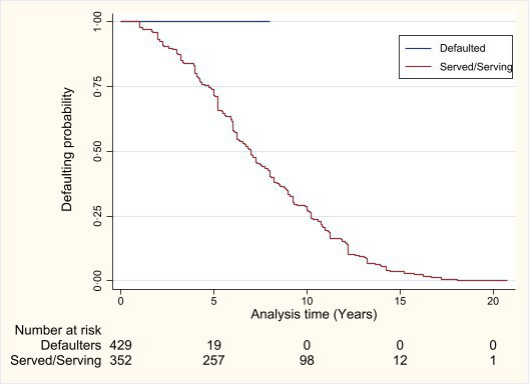
Kaplan-Meier survival estimates for South African RoS scheme beneficiaries. RoS, return-of-service.

A total public sector service duration of 3507.5 person years was observed during the 22.2 years of follow-up, and the longest observed exit is 20.8 years (median=7.2 years; (p25–p75: 5.2–10.6 years)) of public health sector service. Those who defaulted were more likely to not serve at all, hence the flat line in figure 3.

Table 4 shows that at any time during the follow-up, those who studied medicine in South Africa had a 2.6 times higher likelihood of defaulting (HR 2.6; 95% CI 1.6 to 4.0) than those who studied in Cuba, and this was statistically significant (p<0.0001).

**Table 4:** Cox-proportional hazard models predicting retention into RoS schemes in South Africa for medicine and other programmes (2001–2023)

Characteristics	Medicine				Programmes other than medicine			
	n	Time at risk (PY)	HR (95% CI)	P value	n	Time at risk (PY)	HR (95% CI)	P value
Age 10*	486	1936.7	0.3 (−0.6 to 0.03)	0.078	283	1536.9	0.1 (−0.3 to 0.6)	0.544
Race; n (%)								
African	453	1805.4	ref	ref	262	1406.3	Ref	ref
Mixed race	26	116.5	0.7 (0.4 to 1.3)	0.258	5	30.0	0.4 (0.1 to 2.6)	0.311
White	11	36.7	1.1 (0.5 to 2.2)	0.795	11	65.8	0.6 (0.2 to 1.8)	0.330
Indian	5	7.0	1.6 (0.5 to 4.9)	0.448	1	20.8	3.0 (0 to .)	1.000
Medicine								
Cuba	83	348.7	ref	ref	–	–	–	–
South Africa	413	1617.9	2.6 (1.6 to 4.0)	<0.0001	–	–	–	–
Other programmes								
Dentistry	–	–	–	–	50	358.0	ref	
Pharmacy	–	–	–	–	141	695.9	1.6 (0.9 to 2.7)	0.079
Physiotherapy	–	–	–	–	37	190.9	1.6 (0.8 to 3.0)	0.185
Occupational therapy	–	–	–	–	40	234.4	1.1 (0.5 to 2.1)	0.882
Speech therapy and/or audiology	–	–	–	–	17	61.5	2.0 (0.9 to 4.2)	0.083

*Ten-year increase in age of completion, coefficient used and not HR.

HR, Hazard Ratio; PY, person-year.

## Discussion

In this study of four Southern African countries, we found that the success of RoS schemes is not only reliant on the quality of the policy and strength of the legal contract but is likely heavily restricted by the quality of data. There was poor coordination between the funding unit and the employing unit or ministry, making it extremely challenging to monitor the retention or defaulting of beneficiaries. We found that while a third of the beneficiaries had not defaulted their contracts, only 24.3% had fully served their contracts; 21.7% (229/1056) served beyond their obligatory period and 20.2% (213/1056) of beneficiaries were still serving their contractual fulfilment. Medical doctors were statistically more likely to default than dentists, and most of those who defaulted never returned to their provinces. In-fact, undertaking internship and community service outside the provinces was associated with a higher risk of not serving their contracts. We found systemic failures in the implementation of RoS Schemes which render these schemes ineffective. While these schemes aim to target individuals with top academic grades in high school, balanced with the need to support those from low socioeconomic groups in South Africa, the lack of data on these indicators prevents this from being evaluated. Furthermore, data on defaulter reimbursements and/or loan recovery were not retrievable. With more than 90% of South African beneficiaries being African, the objective to redress challenges of the past seems to have been incorporated into candidate selection decisions even though it is difficult to assess this without data used in funding decisions (indicators and decision matrices). While the other countries had more female than male beneficiaries, South Africa had less than 50% of female beneficiaries. Except in Lesotho where pharmacists were in the majority, these schemes were biased towards medicine and against allied health professions, such as speech therapy, audiology and, dually qualified speech therapists and audiologists. These complement prior findings that medicine is often the preferred programme driven by the available budget and applicants’ preferences.^3 6^ The higher pharmacy allocations in Lesotho coincide with the introduction of pharmacy in the country’s university, suggesting that allocations were driven by what was available in-country, rather than the needs of the health system.^6^

This study builds evidence about the importance of health system level levers while developing and implementing policies for health workforce strengthening. While at face value, RoS policies seem useful in targeting areas of need, care needs to be taken to think through the governance, information systems, funding mechanisms, ongoing monitoring and evaluation of such schemes.^10^ Such policies should also be accompanied by a risk management strategy at each possible point of attrition.^14^ While literature has traditionally focused on beneficiary push and pull factors as reasons for health systems’ failure to retain beneficiaries, there are governance, financial and policy environment loopholes which enable defaulting.^3 4 7 15^ In the midst of these weaknesses, these countries continue to face critical HRH shortages of health workforce.^16–21^

The finding that RoS schemes are not effective is consistent with literature which has described RoS implementation as being unidimensional.^12 22 23^ The 1.6% academic failure rate in our study is comparable to the 1.0% reported by Australian respondents who were former RoS beneficiaries for their medical studies.^24^ Even though the 24.3% of beneficiaries who fulfilled their contracts is statistically lower than the 36% (n=109/304; p=0.0001) reported for USA medical doctors in 1992, our results add to these findings in demonstrating relatively poor impact.^12 25^ Previous studies’ findings of RoS beneficiaries being less likely to serve in rural areas than those who go there out of choice could prove true for this study as the two South African provinces (NC and MP) with longitudinal data are mostly rural.^12 25 26^ Other studies have found higher retention rates for health professionals who sign service contracts post-training than for undergraduate programmes as individuals’ preferences change over time.^8 27 28^ An example of this is Sri Lanka’s medical specialist programme which has defaulter rates of between 11 and 13% over 29 years, and 69% repayment of funding for defaulters.^29^

Grobler *et al*’s^4^ 2015 systematic review on effective interventions to retain rural health workers, only found one effective intervention for rural and under-served areas, and that was a health system intervention which introduced universal health coverage in Taiwan.^4^ RoS schemes can only be effective if their implementation is informed by evidence-based planning, is streamlined, coordinated and combined with other health workforce recruitment and retention strategies in the context of an effective health system.^2 3 12 22^

The magnitude of the defaulter rate is a major concern raised by this study. More so because a non-government RoS scheme for service in the public health sector in the South African context has yielded a lower defaulter rate of 12.0% (n=10/83)^30^ between 1999 and 2013. This suggests that with correct mechanisms a sizeable proportion of beneficiaries can fulfil their contracts. It might be worth investing some of the funds used for RoS schemes in better supporting the health workforce rather than funding those who then leave (some presumably due to lack of support/opportunities as reported in the Philippines).^26^ It is, therefore, of concern that the schemes are undermined by short-term planning and budgetary shortfalls that leave funded graduates without a post indirectly training health professionals for HICs.^13^ Estimates are that low-income and middle-income country lose as much as US$15.9 billion annually due to doctor migration to HICs.^21^ Even though not an objective of the study, this study indirectly determined governance and policy enablers of poor retention and they are summarised in table 5.

**Table 5:** Governance and policy reasons for ineffectiveness of RoS schemes in Southern Africa

Governance	Policy
Poor coordination between various ministries (education, labour, health) Isolated implementation by different role players with poor coordination Poor information systems that are not interoperable Allocation of schemes not evidence based Manual entry of data (eg, individuals’ details could be mispelt making it easier for them to default)	Internship outside funding province prolongs exposure to other environments. Internship not counting towards obligatory service makes it easier for beneficiaries to explore other opportunities. The fact that beneficiaries could default during community service shows that the application system does not have enough systems to restrict beneficiaries from not returning to their province. Lack of a risk management plan.

RoS, return-of-service.

The 65.1% (229/352) of beneficiaries who served beyond their contractual obligation indicates that, while RoS schemes may have had some effect, many of these 229 individuals may have chosen to work in their home areas in the public health sector of their own volition regardless of an RoS obligation. This, therefore, raises a question regarding the degree of value added by RoS schemes in retention of health professionals in underserved areas, relative to, for example, targeted admission criteria at universities that favour students of rural origin.

The implementation of RoS schemes needs a whole of government approach with better governance framework, good data management and policy amendment. The higher rate of defaulters associated with internship undertaken outside the funding province calls for a change in policy and practice enabling students to undertake an internship in the province where they were funded. RoS schemes need to be interconnected between the department of education, health, social services and bundled with other workforce retention strategies.^11^ The skills-mix of selected and allocated beneficiaries must be informed by evidence found in the province or country’s needs assessment. Information systems need to be strengthened with good quality data and digital systems must be interoperable with other relevant information systems. Subnational (eg, provincial, district) health systems have to build adequate internal capacity to absorb all RoS beneficiaries from their internship period (eg, match beneficiary allocations with internal internship training capacity, increase internship training platform by strengthening hospital supervision and health sciences training institutions). RoS beneficiaries have to undertake an internship within their funding province (similar to the aboriginal medical scholarship scheme model)^31^ even if this period does not count towards their service obligation. Lastly, RoS beneficiaries have to be preallocated a service area, for example, being linked to the smallest subnational level of governance (eg, subdistrict) and by default a health facility supporting that subdistrict to ensure better planning for future positions and salaries, and better monitoring of beneficiaries' compliance with their service obligations. It is well possible that there might be other strategies and approaches that would be effective depending on the context (structural health system or educational system factors), and those would also need to be incorporated as deemed fit.

The study has several limitations mostly arising from the retrospective nature of the study design. Researchers relied on secondary data from Microsoft Excel databases which could have resulted in misclassification of some beneficiaries’ details, for example, programme of study. This is unlikely to have resulted in bias as all those who were employed by their provinces were found to have been allocated to the correct programme. The absence and poor quality of the data (eg, as reflected in online supplemental appendix C) is a major limitation of this study which could have inflated or lowered the defaulter rate with unknown impact on the estimates of effectiveness found here. For instance, in the Eastern Cape province which only reported on medical students who were funded to study in Cuba but could not retrieve data of beneficiaries who studied within South Africa for the entire period. There is a possibility that the defaulter rate of the combined samples could have been different. However, the absence of data is a finding in itself as it limits the monitoring and tracing of beneficiaries. The number of full records retrieved (1116) is 12.9% less than the calculated minimum sample size. This is unlikely though to have affected the reliability of these findings as the sample size calculated was 1067 and it factored for attritions and data entry errors to arrive at the minimum desired sample size of 1281. The retrospective nature of the study made it impossible to adjust for confounders due to lack of socioeconomic details, and inability to triangulate with university databases and beneficiaries. The study is further limited by the inability of the payroll system to monitor individuals beyond their province even if they worked elsewhere in the public health sector. Furthermore, in the absence of costing data, positive results of the Cuban medical programme need to be interpreted with caution. Notwithstanding, this study still provides the best available evidence for the effectiveness of government-run RoS schemes in the four countries.

10.1136/bmjgh-2023-013687.supp3 Supplementary data

## Conclusion

Evaluation of the RoS schemes and likely the schemes’ effectiveness is hindered by poor data management of beneficiary records. Of the small proportion of beneficiaries with full-service records (19.9%), full return of service could be demonstrated for only a quarter of beneficiaries representing a waste of limited resources. Internship training within the fnding province was found to play a significant role in the effectiveness of these schemes. A whole of government approach is needed to improve the implementation of RoS schemes and strengthen the health workforce in these countries. This study recommends further research on RoS schemes including the cost-effectiveness and return on investment of these schemes.

## Data Availability

Data are available on reasonable request. Deidentified data used in this study are available from the corresponding author on reasonable request.
