# Supplementary material for: Effectiveness of return-of-service schemes for human resources for health retention: a retrospective cohort study of four Southern African countries
Source: BMJ Glob Health. 2023 Oct 24;8(10):e013687. doi: 10.1136/bmjgh-2023-013687 (PMC10603424; doi:10.1136/bmjgh-2023-013687)
Supplement: Supplementary data [file bmjgh-2023-013687supp001.pdf]

Appendix A: Access Database

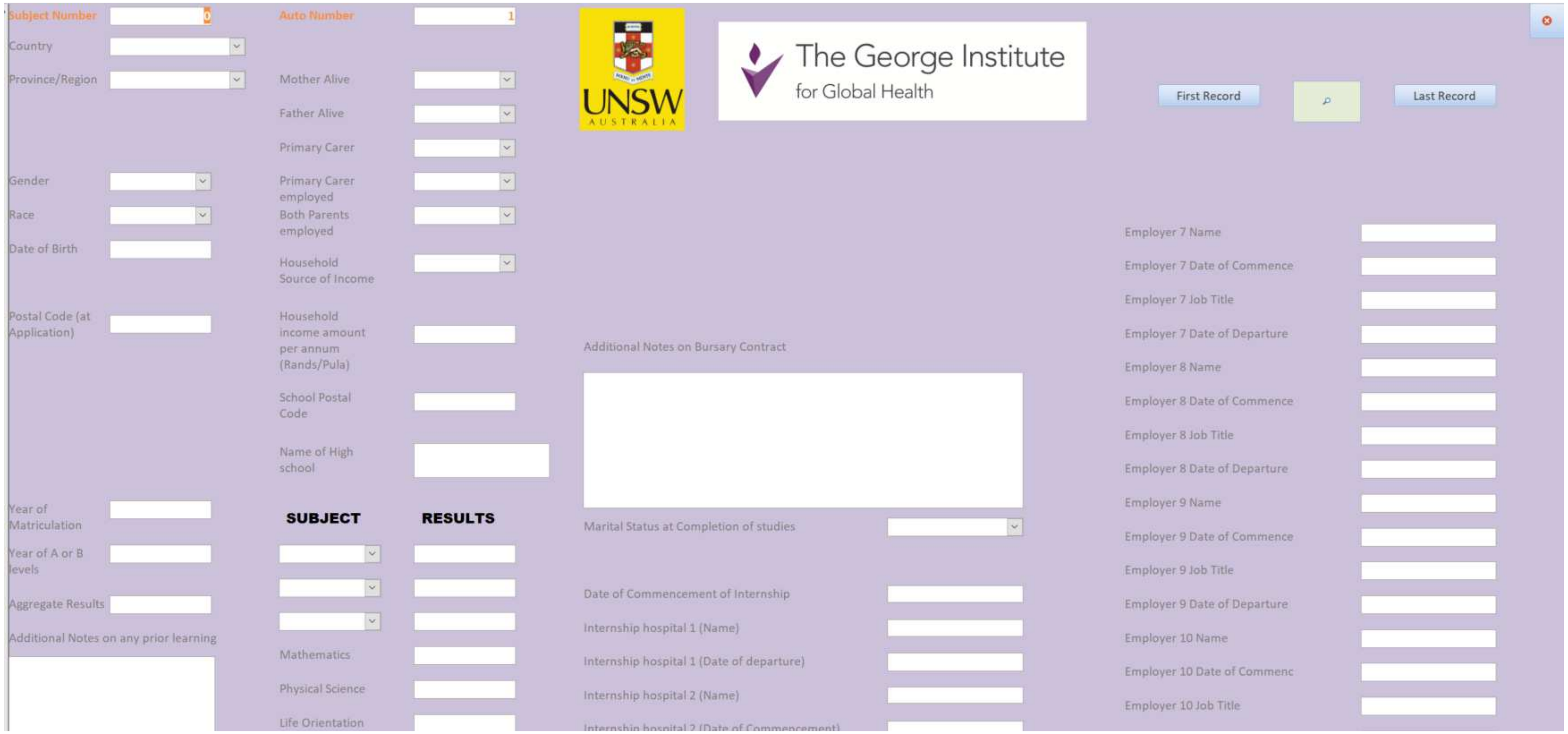

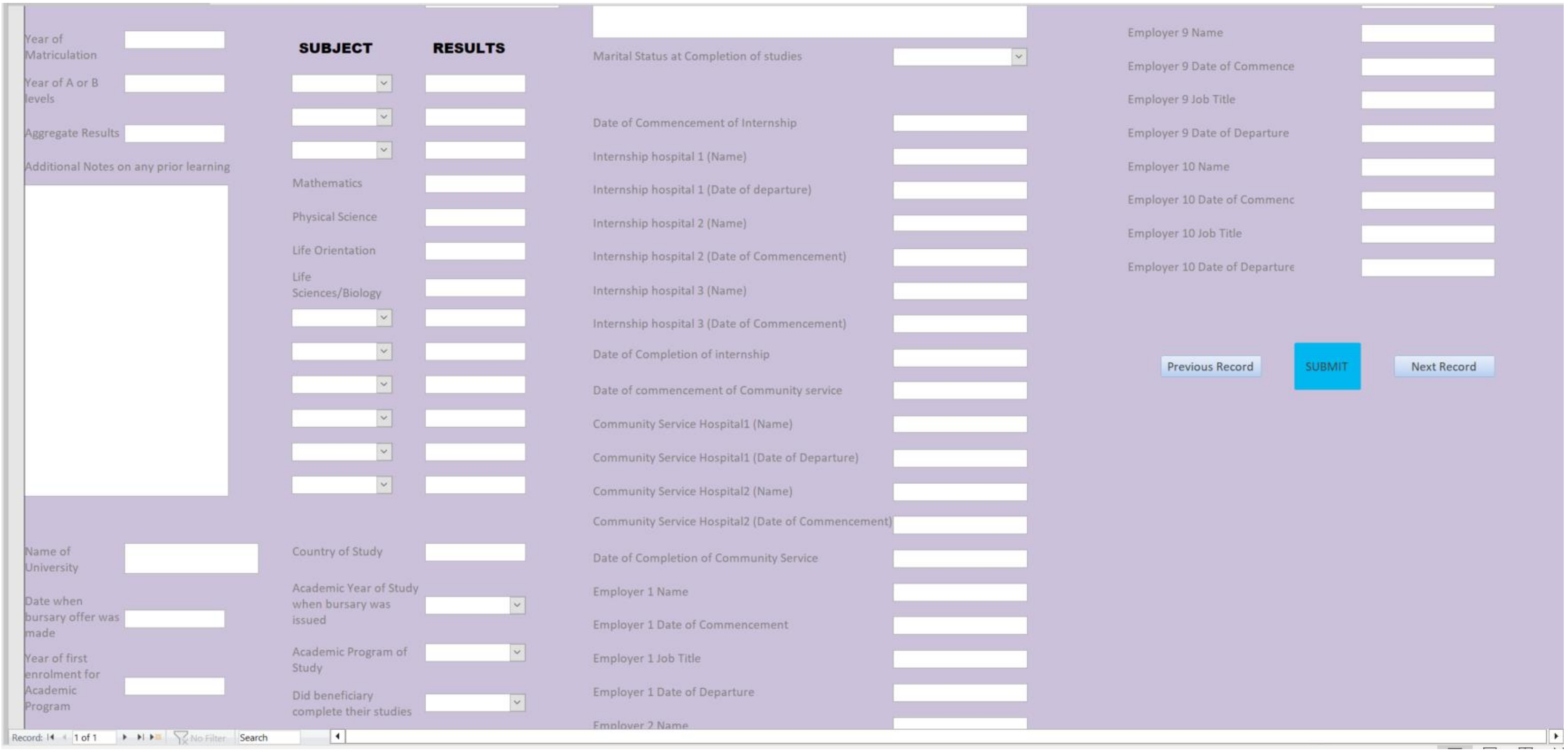
Year of Matriculation
Year of A or B levels
Aggregate Results
Additional Notes on any prior learning
SUBJECT
RESULTS
Mathematics
Physical Science
Life Orientation
Life Sciences/Biology
Name of University
Date when bursary offer was made
Year of first enrolment for Academic Program
Country of Study
Academic Year of Study when bursary was issued
Academic Program of Study
Did beneficiary complete their studies
Marital Status at Completion of studies
Date of Commencement of Internship
Internship hospital 1 (Name)
Internship hospital 1 (Date of departure)
Internship hospital 2 (Name)
Internship hospital 2 (Date of Commencement)
Internship hospital 3 (Name)
Internship hospital 3 (Date of Commencement)
Date of Completion of internship
Date of commencement of Community service
Community Service Hospital1 (Name)
Community Service Hospital1 (Date of Departure)
Community Service Hospital2 (Name)
Community Service Hospital2 (Date of Commencement)
Date of Completion of Community Service
Employer 1 Name
Employer 1 Date of Commencement
Employer 1 Job Title
Employer 1 Date of Departure
Employer 9 Name
Employer 9 Date of Commence
Employer 9 Job Title
Employer 9 Date of Departure
Employer 10 Name
Employer 10 Date of Commenc
Employer 10 Job Title
Employer 10 Date of Departure
Previous Record
SUBMIT
Next Record
Record: 1 of 1
No Filter
Search

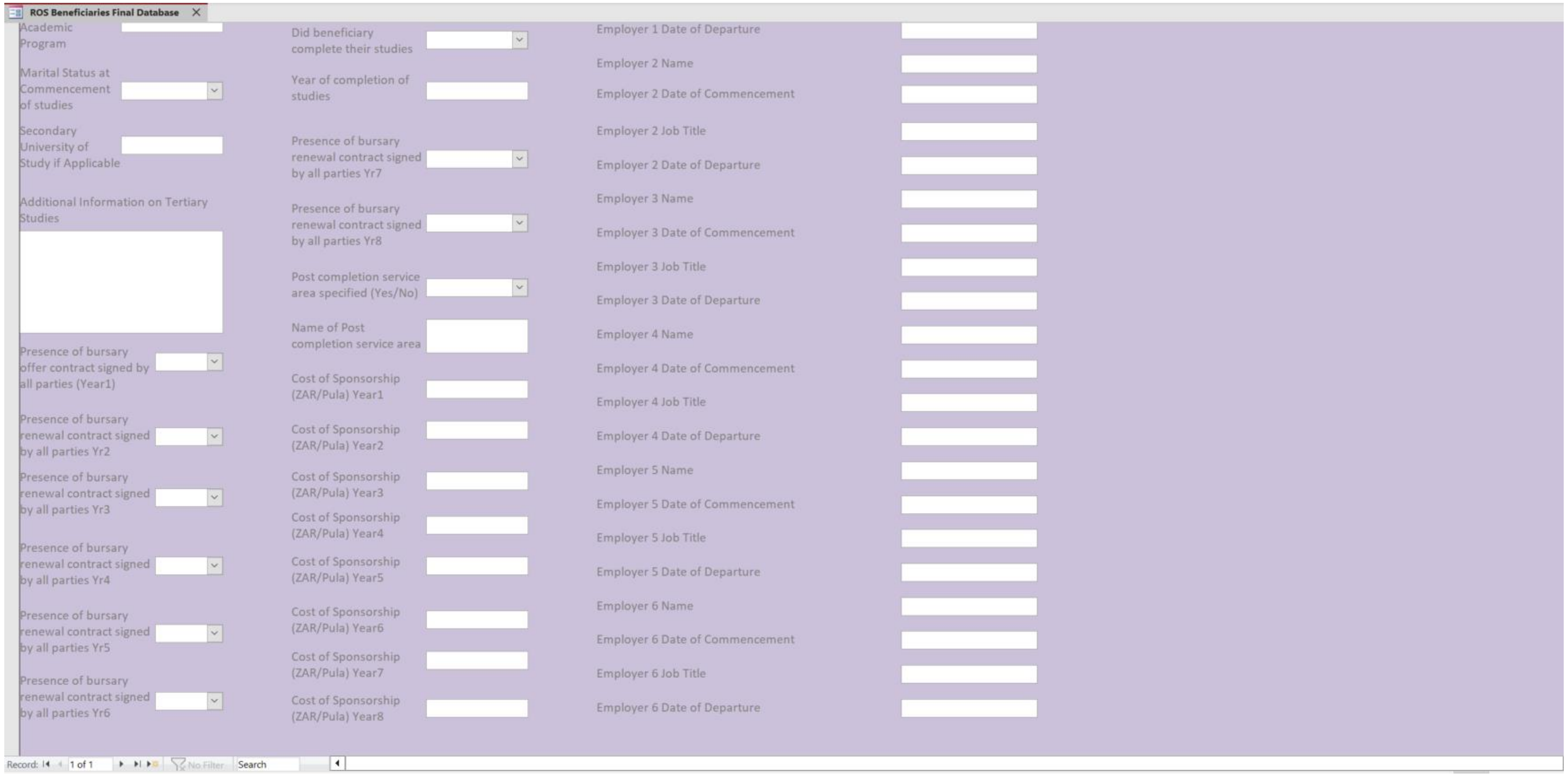
ROS Beneficiaries Final Database

Academic Program

Marital Status at Commencement of studies

Secondary University of Study if Applicable

Additional Information on Tertiary Studies

Presence of bursary offer contract signed by all parties (Year1)

Presence of bursary renewal contract signed by all parties Yr2

Presence of bursary renewal contract signed by all parties Yr3

Presence of bursary renewal contract signed by all parties Yr4

Presence of bursary renewal contract signed by all parties Yr5

Presence of bursary renewal contract signed by all parties Yr6

Did beneficiary complete their studies

Year of completion of studies

Presence of bursary renewal contract signed by all parties Yr7

Presence of bursary renewal contract signed by all parties Yr8

Post completion service area specified (Yes/No)

Name of Post completion service area

Cost of Sponsorship (ZAR/Pula) Year1

Cost of Sponsorship (ZAR/Pula) Year2

Cost of Sponsorship (ZAR/Pula) Year3

Cost of Sponsorship (ZAR/Pula) Year4

Cost of Sponsorship (ZAR/Pula) Year5

Cost of Sponsorship (ZAR/Pula) Year6

Cost of Sponsorship (ZAR/Pula) Year7

Cost of Sponsorship (ZAR/Pula) Year8

Employer 1 Date of Departure

Employer 2 Name

Employer 2 Date of Commencement

Employer 2 Job Title

Employer 2 Date of Departure

Employer 3 Name

Employer 3 Date of Commencement

Employer 3 Job Title

Employer 3 Date of Departure

Employer 4 Name

Employer 4 Date of Commencement

Employer 4 Job Title

Employer 4 Date of Departure

Employer 5 Name

Employer 5 Date of Commencement

Employer 5 Job Title

Employer 5 Date of Departure

Employer 6 Name

Employer 6 Date of Commencement

Employer 6 Job Title

Employer 6 Date of Departure

Record: 1 of 1 No Filter Search
