# Supplementary material for: Effectiveness of return-of-service schemes for human resources for health retention: a retrospective cohort study of four Southern African countries
Source: BMJ Glob Health. 2023 Oct 24;8(10):e013687. doi: 10.1136/bmjgh-2023-013687 (PMC10603424; doi:10.1136/bmjgh-2023-013687)
Supplement: Supplementary data [file bmjgh-2023-013687supp002.pdf]

Appendix B: Survival analysis models

Model 1: Beneficiaries other than Medical Beneficiaries

**Model no**	**Model**	**Nested P-value (chi)^2**	**AIC**
EE	Programme[♣]	<0.0001	1182.39
HH	Programme[♣] + Age**	<0.0001	1159.295
II[#]	Programme[♣] + Age** + Race	<0.0001	1144.392
[♣]Beneficiaries other than Medical Beneficiaries; **Age at completion of studies; [#]Best Model overall.			

Model 2: Medical Beneficiaries

**Model no**	**Model**	**Nested P-value (chi)^2**	**AIC**
DD	Programme[⁎]	<0.0001	1833.15
LL	Programme[⁎] + Age**	<0.0001	1789.837
PP[#]	Programme[⁎] + Age** + Race	<0.0001	1786.289
[⁎]South African vs Cuban based medical beneficiaries; **Age at completion of studies; [#]Best Model overall.			
