# Supplementary material for: Effectiveness of return-of-service schemes for human resources for health retention: a retrospective cohort study of four Southern African countries
Source: BMJ Glob Health. 2023 Oct 24;8(10):e013687. doi: 10.1136/bmjgh-2023-013687 (PMC10603424; doi:10.1136/bmjgh-2023-013687)
Supplement: Supplementary data [file bmjgh-2023-013687supp003.pdf]

Appendix C: Validity of Databases' Quality

To ascertain data validity, provinces and countries were given a template to submit aggregate data per programme for each year of assessment (2000-2010). Programmes were only limited to those assessed in this study, namely; Medicine, Pharmacy, Dentistry, Physiotherapy, Occupational Therapy, Speech Therapy, Audiology and, Audiology and Speech Therapy dual qualification. These aggregate data were checked against the individual level data to confirm the equality of these data. However, only Limpopo province and KwaZulu-Natal province in South Africa sent these aggregate level data. Whilst aggregate data should be a summation of individual level data, this does not always seem to be the case in practice. Policymakers might for instance only be interested on reporting the aggregate number of funded beneficiaries in a given year and not capture some of their individual level characteristics. It's also possible for either form of the data to not be retrievable after some time if the database quality is poor. As shown in Figure 1, KwaZulu Natal province reported 5.7, 5.1, 4.6, 3.9, 3.6 and 3.1 times more aggregated beneficiaries than the captured individual level beneficiaries for Pharmacy, Physiotherapy, Occupational Therapy, Dentistry, Speech Therapy and/or Audiology, and Medicine respectively.

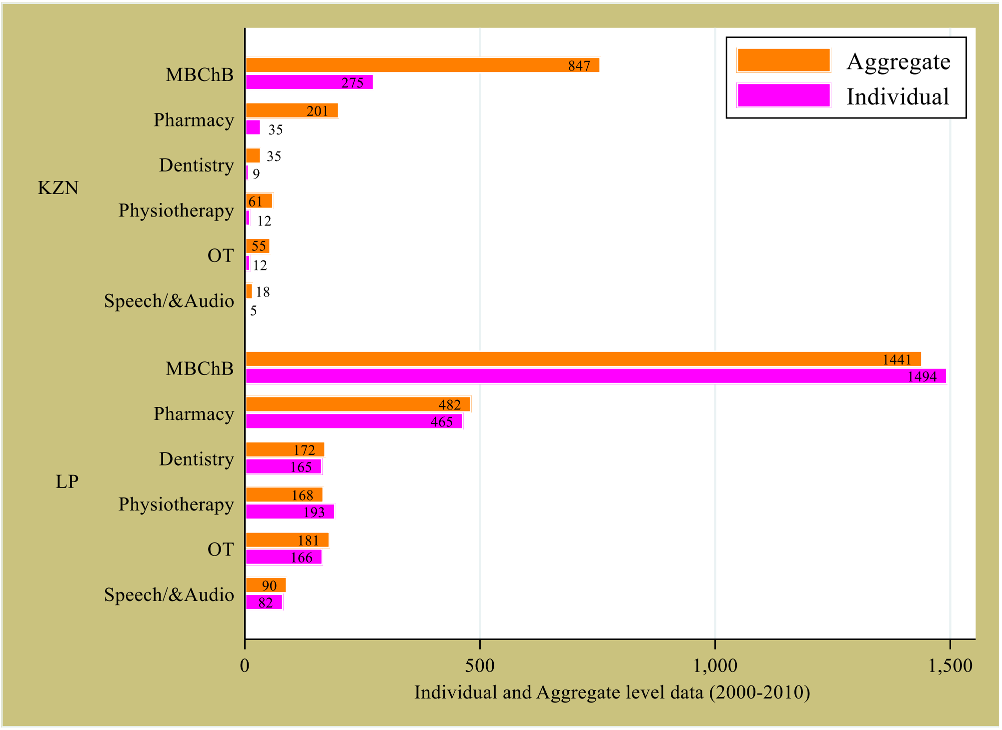

Figure 1: Aggregate and Individual level data in two South African Provinces (2000-2010)
KZN=KwaZulu Natal province; LP=Limpopo province; MBChB=Bachelor of Medicine and Surgery; OT= Occupational Therapy; Speech/&Audio=Speech Therapy and/or Audiology

Figure 1 further shows that whilst Limpopo province reported almost equal individual level and aggregate level beneficiaries for all the programmes, Physiotherapy and Medicine reported more individual level beneficiaries. All other programmes reported slightly more aggregated level beneficiaries.

Figure 2 and Table 1 show that whilst KwaZulu-Natal province could not retrieve individual level data for 2003-2006, there were 159 medicine beneficiaries reported at the individual level.

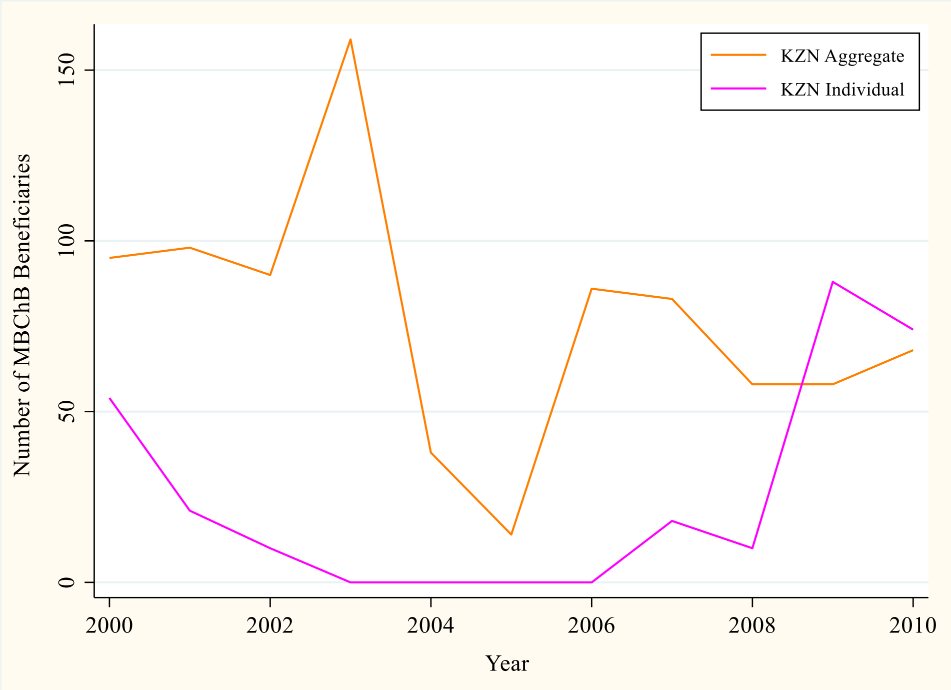

Figure 2: Aggregate and Individual level Medicine beneficiaries' data KwaZulu-Natal (2000-2010)
KZN=KwaZulu Natal province; MBChB=Bachelor of Medicine and Surgery

Table 1 further shows that Limpopo province's data did not show statistically significant differences between individual and aggregate level data (p-value >0.05). In contrast, all but one data comparison (dentists) showed statistically significant differences (p<0.05) between individual and aggregate level data in KwaZulu-Natal province.

Table 1: Aggregate and Individual level data in two South African Provinces (2000-2010)

Prov	Year	MBChB			Pharmacy			Dentistry			Physio			OT			Speech&/Audio		
		Agg	Indi	p-value	Agg	Indi	p-value	Agg	Indi	p-value	Agg	Indi	p-value	Agg	Indi	p-value	Agg	Indi	p-value
	2000	71	71		11	11		1	1		9	9		17	15		1	1	
	2001	62	63		9	9		3	3		4	4		7	9		13	13	
	2002	232	256		36	36		40	40		18	18		17	17		3	3	
	2003	47	46		10	10		2	2		1	1		4	4		1	1	
	2004	55	70		23	24		4	4		1	4		1	0		1	1	
LP	2005	131	177	0.098	77	118	0.625	13	19	0.403	25	26	0.250	5	8	0.633	1	1	0.500
	2006	166	198		40	40		16	20		8	25		11	9		16	13	
	2007	177	185		86	63		25	26		12	32		18	15		7	7	
	2008	221	230		108	108		27	27		35	35		65	65		20	20	
	2009	143	166		44	44		18	18		36	36		18	21		17	17	
	2010	136	32		38	2		23	5		19	3		18	3		10	5	
	Total	1441	1494		482	465		172	165		168	193		181	166		90	82	
	2000	95	54		12	0		13	0		11	0		5	0		2	0	
	2001	98	21		10	0		3	0		5	0		2	0		3	0	
	2002	90	10		24	0		0	0		0	0		3	0		0	0	
	2003	159	0		0	0		0	0		0	0		0	0		0	0	
	2004	38	0		16	0		5	0		0	0		2	0		1	0	
	2005	14	0		13	0		1	0		4	0		3	0		3	0	
KZN	2006	86	0	0.001	10	0	0.002	3	0	0.117	0	0	0.016	7	0	0.004	1	0	0.031
	2007	83	18		41	0		4	0		9	0		11	0		2	0	
	2008	58	10		15	0		4	0		7	0		8	0		2	0	
	2009	58	88		28	19		0	6		7	6		8	6		2	2	
	2010	68	74		32	16		2	3		18	6		6	6		2	3	
	Total	847	275		201	35		35	9		61	12		55	12		18	5	

Agg=aggregate data; Indi=individual data; Prov = Province; LP=Limpopo province; KZN=KwaZulu-Natal province; MBChB=Bachelor of Medicine and Surgery (Medicine); Physio=Physiotherapy; OT=Occupational Therapy; Speech&/Audio=Speech therapy, Audiology or dually qualified Speech Therapist and Audiologist.

The Wilcoxon signed-ran test was used to compute p-values.
